# Unilateral Epididymo-Orchitis With Complicated Urinary Tract Infection: A Clue to Underlying Vesicoureteral Reflux

**DOI:** 10.7759/cureus.85160

**Published:** 2025-05-31

**Authors:** Rajarajan Paulpandian, Upasana Ranga

**Affiliations:** 1 Pediatrics/Neonatology, Apollo Hospitals, Chennai, IND; 2 Radiology, Apollo Hospitals, Chennai, IND

**Keywords:** complicated uti, epididymo-orchitis, infant, torsion testis, vesicoureteral reflux

## Abstract

Epididymo-orchitis (EO) is rare in infants, and associated hydrocele at presentation indicates an advanced stage of infection. It is commonly associated with urinary tract infection (UTI) and can be a clue to the presence of underlying congenital anomalies of the kidney and urinary tract. Testicular torsion is a surgical emergency that can mimic EO and should be ruled out at presentation. Here, we present a six-month-old male infant who was brought in with complaints of passage of pinkish urine, pus discharge per urethra for two days, and fever for one day. He also had a history of excessive crying during micturition for the past seven days. On examination, he was febrile, and head-to-toe examination revealed an acute left scrotum with hydrocele. Prehn’s sign was negative on the left side, and the cremasteric reflex was absent bilaterally. Urinalysis was ordered along with culture, which showed plenty of pus cells and a few red blood cells. Ultrasound with Doppler (USG Doppler) was done immediately, which ruled out torsion and hernia but showed an inflamed epididymis and left mild hydronephrosis (HDN) with trabeculated bladder wall and internal echoes. The infant was started on intravenous (IV) antibiotics for suspected UTI with poor oral intake, and he promptly improved in the next 48 hours. Urine culture grew *Escherichia coli, *and an antibiotic course was given for 10 days. A follow-up micturating cystourethrogram (MCU) revealed grade III left side vesicoureteral reflux (VUR), for which he was started on antibiotic prophylaxis, and his technetium-99m dimercaptosuccinic acid (Tc-99m DMSA) scan was normal. He is currently under periodic follow-up for his VUR. This case emphasizes the timely detection of complicated UTIs in infants. Torsion should always be ruled out in infants presenting with acute scrotum.

## Introduction

Epididymo-orchitis (EO) is one of the common causes of pediatric acute scrotum. The exact incidence is difficult to establish with studies reporting a wide variation ranging between 3.7% and 71% of children presenting with acute scrotum [1]. It has a bimodal distribution peaking in infancy and puberty [2]. Etiology can be divided into infectious and noninfectious. Infectious etiology varies between infants and older children. Viral infections and postviral inflammation are presumed to be the main etiology in older children [3]. It is defined by the absence of pyuria. Mumps was the main causative agent before the advent of widespread vaccination. Currently, other viruses like coxsackievirus A, echovirus, and varicella have also been identified. Somekh et al. found that only one out of 44 children with EO had a urinary tract infection (UTI), and 20% of them had serological evidence for recent viral infection [4].

Bacteremia as a result of sepsis seems to be the etiology in neonates [5]. Congenital anomalies of the kidney and urinary tract (CAKUT) are the main causes of EO in infants and younger children. The retrograde flow of infected urine through the vas deferens to the epididymis and testis can cause EO. Common associations have been CAKUT like vesicoureteral reflux (VUR), posterior urethral valve, ectopic ureter, ectopic vas deferens, imperforate anus, and neurogenic bladder [6]. Functional voiding problems can also cause EO in children. Coliform organisms like* Escherichia coli*, *Klebsiella*, *Proteus*, and *Pseudomonas *are the commonly implicated bacterial organisms in UTI as well as EO [6]. Noncoliforms like tuberculosis and brucellosis are rare bacterial causes of EO in older children. Brucellosis should be suspected when a child, especially one who has a history of consuming unpasteurised dairy products or contact with farm animals, presents with symptoms like fever, arthralgia, hepatosplenomegaly, along with EO. Tuberculosis is present throughout the world, with the highest burden seen in Southeast Asia, Africa, and the Western Pacific. It spreads hematogenously to the gonads and can cause symptoms resembling a tumorlike caseous necrosis, testicular enlargement, and scrotal ulcers [7,8]. 

Noninfectious etiology of EO includes torsion of the testis, torsion of the testicular appendage and incarcerated hernia, and systemic diseases like Henoch-Schönlein purpura (HSP)[8]. Torsion testis is a surgical emergency, as a delay in diagnosis can affect the viability of the testis, and so is an incarcerated hernia. Torsion of the gonad appendages is a self-limiting mechanical cause of inflammation, which can be easily diagnosed by physical examination and Doppler ultrasound (USG Doppler) (characteristic “blue dot sign”) and resolves with conservative management. In one review of 238 consecutive boys, ages 0 to 19 years, who presented with acute scrotal pain to a children's hospital over a two-year period, 16% had testicular torsion, 35% had epididymitis, and 46% had torsion of the appendix testis [9]. We report this case to highlight the importance of the timely management of complicated UTI in infants and the need to rule out UTI in infants upfront when they present with acute EO.

## Case presentation

**Figure 1:**
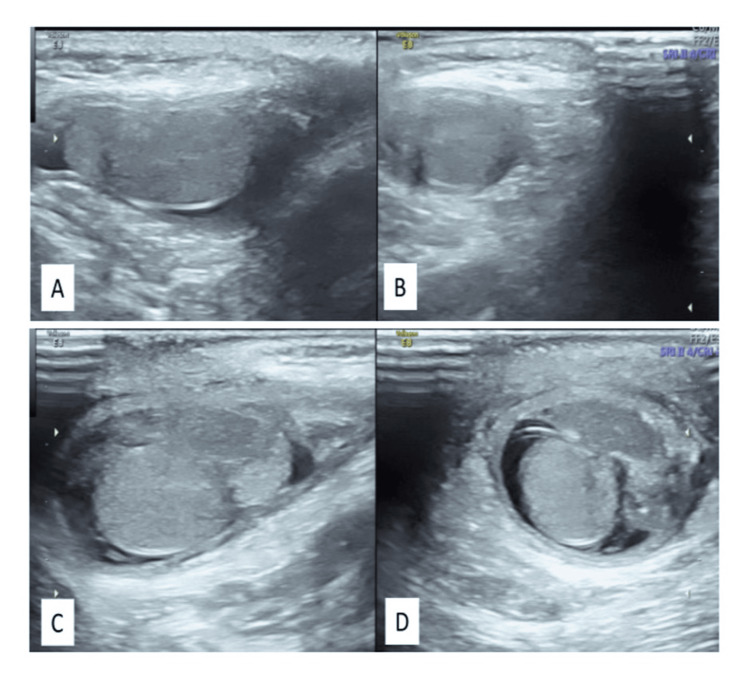
Ultrasound images of the right and left scrotal sacs showing mildly bulky left epididymis and testis (C, D) when compared to the right side (A, B). Mild hydrocele is also seen in both sides

**Figure 2:**
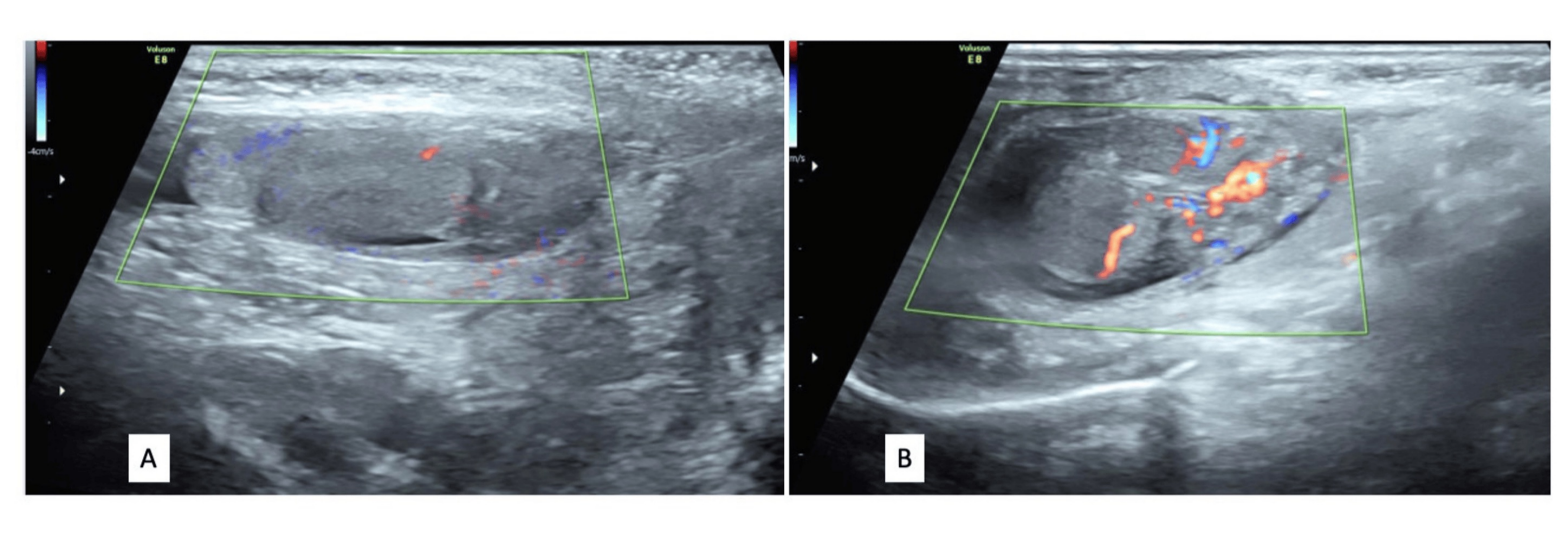
Color doppler ultrasound images of the right (A) and left (B) scrotal sacs showing increased vascularity of the left epididymis and testis

A six month male infant was brought with complaints of passing altered colour urine (pink) and a pus like discharge noticed per urethra for two days and fever for one day. The infant was also passing urine more frequently for a week prior to admission. On examination, he was febrile (38°C) and found to be slightly irritable. A urine routine along with culture was sent through catheterisation suspecting UTI, which showed plenty of pus cells and few red blood cells. While we were reassessing him for his irritability, we found that his left scrotum looked strikingly red and swollen with features of inflammation like warmth, tenderness and alteration in rugosities. Testis was palpable in upper part of scrotum and a cord like structure was also palpable along with testis in the upper scrotum which we assumed could be an inflamed epididymis. Trans-illumination test was positive s/o hydrocele. Right scrotum and testis were normal. There was neither a visible swelling in inguinal region nor any features of intestinal obstruction, so possibility of an incarcerated hernia was thought to be less likely. Our first priority was to rule out a testicular torsion in an acute scrotum. Clinically, infant was not having systemic features like vomiting nor significant tenderness while examining his left scrotum and while elevating his left testis, he did not cry (positive Prehn’s sign). Cremasteric reflex was absent bilaterally. USG doppler of the inguino-scrotal region was done immediately, which showed increased blood flow to left testis, epididymis and cord structures which were bulkier compared to the right side and bilateral hydrocele were present with the left more than the right (Figures 1, 2). As we were able to rule out torsion and incarcerated hernia upfront, the diagnosis of left EO for the acute scrotum was strongly considered. The next step was to confirm the diagnosis of UTI as the underlying etiology, for which urine culture was sent. 

**Figure 3:**
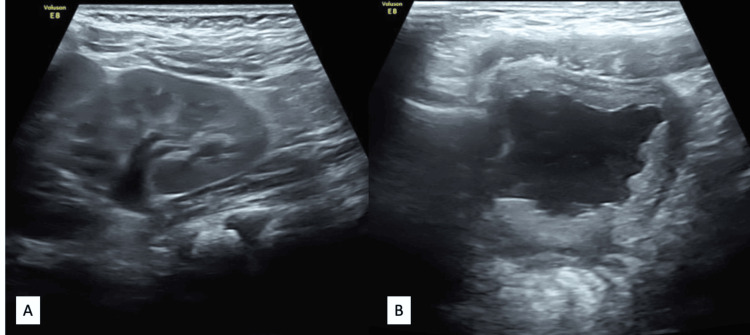
Ultrasound images of (A) the left kidney showing mild hydronephrosis and (B) the bladder showing diffuse wall thickening, trabeculations, and low level internal echoes in the bladder lumen

The infant was admitted with a provisional diagnosis of UTI with acute EO. He was started on intravenous (IV) antibiotics, piperacillin-tazobactam (80 mg/kg/dose thrice a day) and amikacin (15 mg/kg/day as a single dose), as his oral intake was poor. Oral paracetamol was given for fever and pain along with testicular elevation. Blood culture, complete blood count, and C-reactive protein (CRP) were sent as part of the septic workup, along with renal function test (RFT) and electrolytes. Leucocytosis with neutrophilic predominance and elevated CRP were present with normal RFT and electrolytes (Table 1). He started to show prompt improvement with defervescence of fever and decrease in irritability and inflammation of the left scrotum within 48 hours of admission. Meanwhile, urine culture grew *Escherichia coli* by 72 hours, which was sensitive to antibiotics (Table 1). Ultrasound of the kidney, ureters, and bladder (USG KUB) was done, which showed left mild hydronephrosis (HDN) with anteroposterior diameter of 10 mm and trabeculated bladder wall with moving echoes (Figure 3).

**Table 1:** Laboratory findings of the index case CFU: colony-forming unit

Test	Result	Reference range
White blood cells (WBC)	20 x 10^9^/L	6-14 x 10^9^/L
Neutrophils	82%	54-62%
Lymphocytes	13%	25-33%
Hemoglobin (Hb)	13 g/dL	10.5-14 g/dL
Platelet	400 x 10^9^/L	84-478 x 10^9^/L
C-reactive protein (CRP)	31 mg/L	0.8-11.2 mg/L
Urea	15 mg/dL	5-18 mg/dL
Creatinine	0.4 mg/dL	0.03-0.50 mg/dL
Sodium	136 mmol/L	134-144 mmol/L
Potassium	4 mmol/L	3.5-5.6 mmol/L
Chloride	100 mmol/L	98-106 mmol/L
Urine culture (catheterised sample)	Escherichia coli (> 10^4^ CFU*/mL) sensitive: piperacillin-tazobactam, amoxicillin-clavulanate, amikacin, and meropenem; resistant: ceftriaxone, cefotaxime, ceftazidime, aztreonam	
Blood culture	No growth	

**Figure 4:**
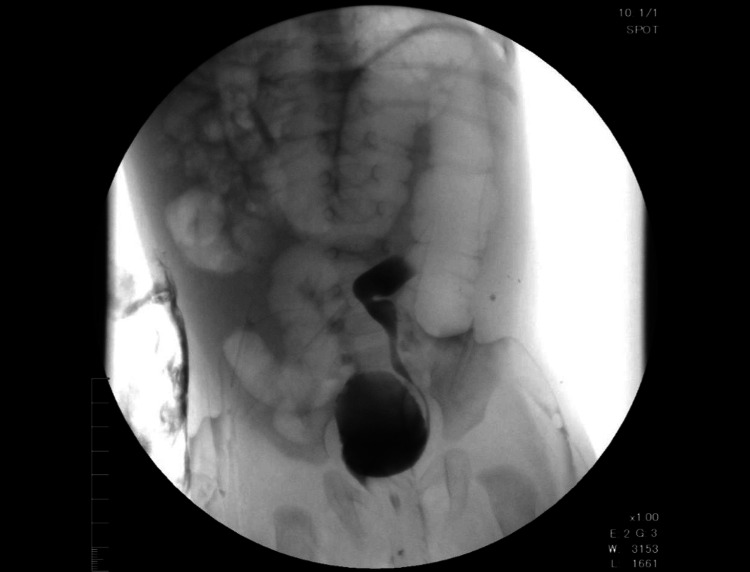
Micturating cystourethrogram (MCU) scan showing left-side grade III vesicoureteral reflux (VUR)

**Figure 5:**
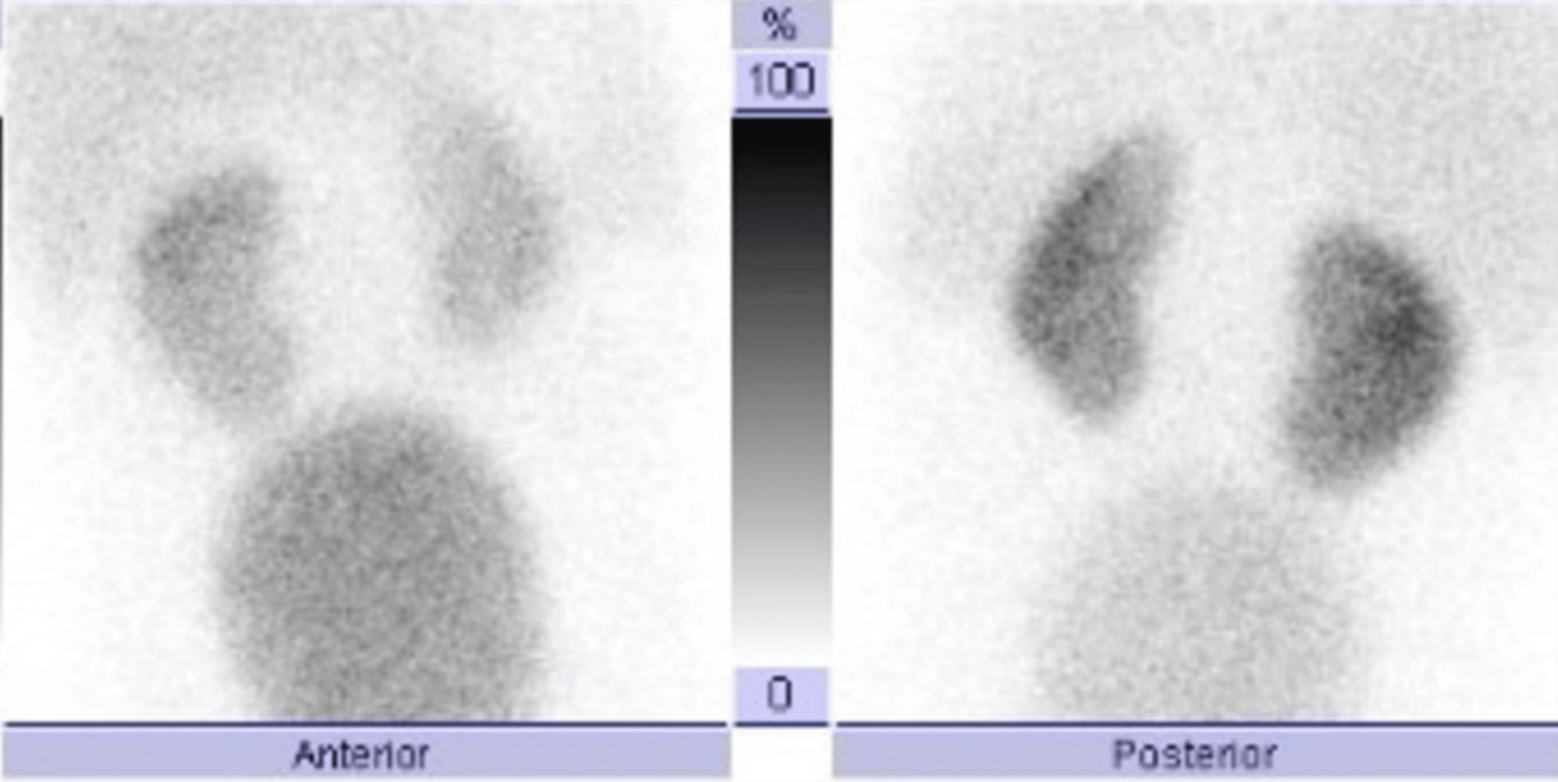
Technetium-99m dimercaptosuccinic acid scan (Tc-99m DMSA) anterior and posterior planar views showing normal uptake in the right and left kidneys without scar

At the end of three days of IV antibiotics, as the infant had become asymptomatic and urine culture with sensitivity was reported, we de-escalated the IV antibiotic to oral amoxycillin-clavulanate (20 mg/kg/dose twice a day) which was given for seven more days (a cumulative course of 10 days) and advised follow up with pediatric nephrology for further evaluation. Follow-up imaging included a micturating cystourethrogram (MCU) scan done four weeks after the episode, which revealed left-side grade III VUR (Figure 4) and a Tc-99m DMSA scan done six months after the episode, which showed normal uptake in both the kidneys with no scars (Figure 5). He was started on antibiotic prophylaxis for grade III VUR with oral nitrofurantoin 1 mg/kg/day (after a normal glucose-6-phosphate dehydrogenase (G6PD) assay) and is being followed up periodically.

## Discussion

Diagnosis of acute EO in an infant can be challenging, as infants can be irritable for many reasons, and unlike in testicular torsion, florid features of acute inflammation can be absent at presentation, which both parents and physicians can miss initially, as what happened in our case. The general dictum is that whenever an infant presents with acute scrotum, testicular torsion should be ruled out [10]. Testicular torsion is a surgical emergency where every minute counts, as a delay in diagnosis can affect the viability of the testis. Testicular torsion has a bimodal peak in incidence, a small percentage in newborns, and another in pubertal age, though it can present in any age [2]. Certain clinical features can help differentiate EO from testicular torsion. EO has positive Prehn's sign and cremasteric reflex, while these are negative in testicular torsion. USG Doppler shows increased blood flow in EO vs absent flow in torsion. But the major point to be noted is that none of these are sensitive enough in picking a torsion. Prehn’s sign is generally considered not a reliable sign in differentiating torsion from EO. Though cremasteric reflex is absent in torsion and preserved in EO, it can generally be absent up to six months of age (as in our case) [11].

The USG Doppler has a sensitivity and specificity of around 89% and 100% in the detection of testicular torsion [12]. The classical features suggestive of torsion are a decrease in testicular perfusion and twisting of the spermatic cord. However, its utility is limited in small prepubertal testes with lower blood flow. Furthermore, scrotal USGs may occasionally show present but diminished blood flow to the testis and epididymis, or even increased blood flow to the epididymis as the result of reperfusion if a testis has either spontaneously or manually detorsed. Hence, the importance of a careful history and physical examination, as well as a clear understanding of the possible limitations in interpreting imaging studies, cannot be overstated. 

The nuclear scan measures have a higher diagnostic ability than USG Doppler (sensitivity and specificity of around 100% and 97%, respectively) [13]. However, it is limited by its longer duration of procedure and nonavailability at many centers, thus making USG Doppler the test of choice for testicular torsion.

**Figure 6:**
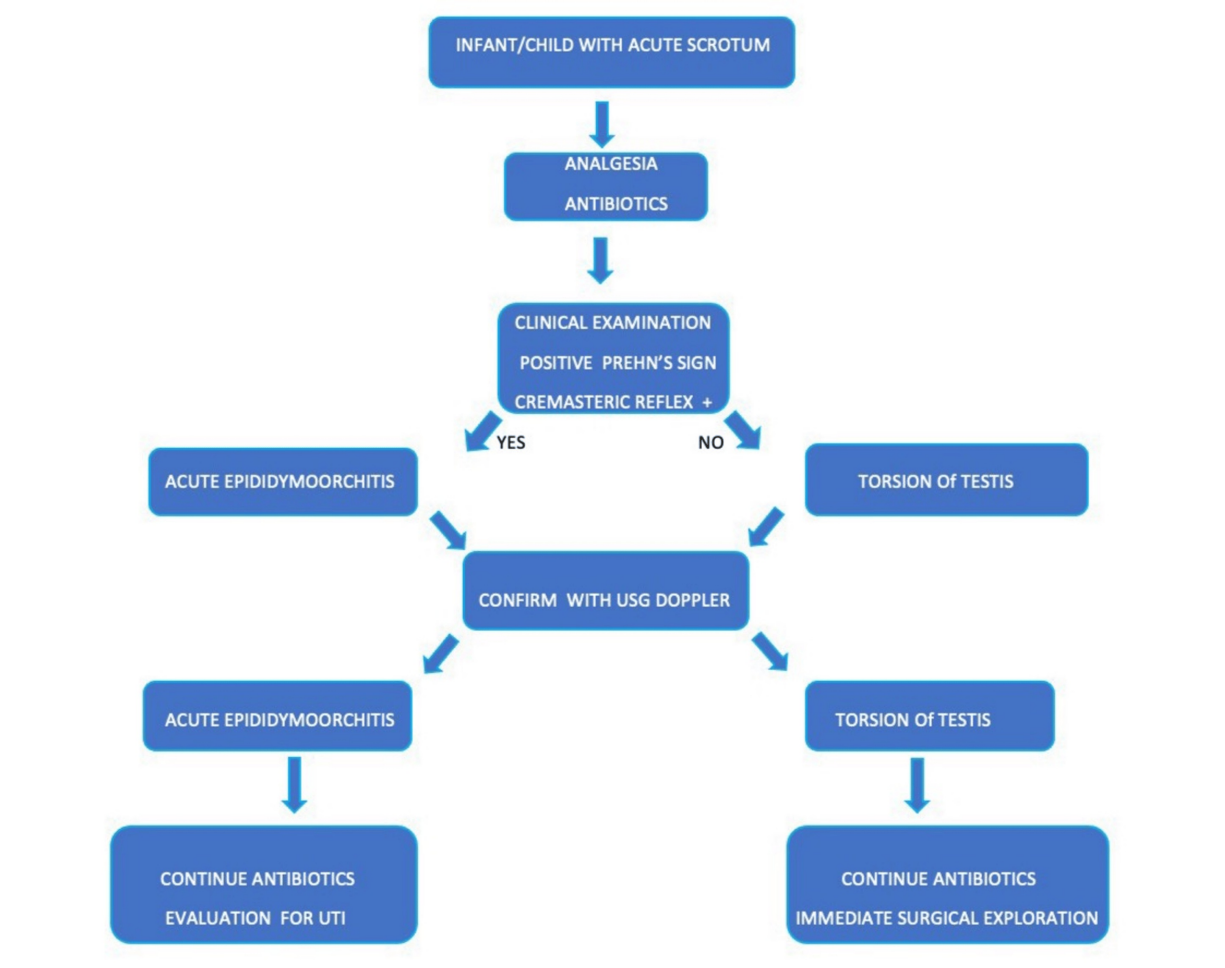
Algorithmic approach to acute scrotum

So, whenever an irritable infant with acute scrotum has nonresolving pain with analgesic and first-dose antibiotic, equivocal USG findings should promptly lead us to a surgical opinion and exploration without wasting much time. At the same time, as EO is more common than torsion testis, a stepwise, timely approach as we followed in our case (Figure 6) will be of great help in differentiating between both and reducing the need for unnecessary surgical exploration and parental anxiety. Torsion of the testicular appendage, a benign and much more common cause of acute scrotum than testicular torsion in children, should also be considered in the differential diagnosis. Incarcerated hernia is another differential, which again is a surgical emergency and should be ruled out upfront using USG [11]. 

The most common cause of EO in infants is secondary to UTI, with coliforms being the commonly implicated organisms [6]. The presence of UTI in infants can cause complications, as there is a strong possibility of underlying CAKUT, which can predispose them to acute pyelonephritis and recurrent UTI. Diagnosis of EO in an infant presenting with pyuria can be a pointer toward underlying CAKUT with UTI, as the retrograde flow of infected urine through the vas deferens causes EO [6]. Septicemia without UTI causing EO has also been reported in newborns and young infants, so blood culture to detect bacteremia should be considered in management, and antibiotic duration should be tailored accordingly [14].

Though EO generally has a benign outcome, major complications like scrotal abscess and testicular necrosis have also been reported in the literature [15]. So, a timely diagnosis and prompt institution of IV antibiotics are a must in newborns and young infants to salvage them from possible sepsis and testicular viability.

The main challenge in the management of such cases in the emergency department will be to differentiate an acute EO from a testicular torsion. The differentiating clinical features and USG Doppler can be of help in such situations, but with the caveat that none of the clinical or USG features are sensitive enough to definitely rule out a testicular torsion. If testicular torsion is strongly suspected, then we should proceed with immediate surgical exploration [16]. The other challenge we faced was in addressing the parental concern regarding the initial possibility of infection/torsion in a genital area in a growing infant. It creates immense stress in them as they worry about the viability of their testis and the future impact on fertility.

## Conclusions

Complicated UTI in infants/children requires prompt initiation of antibiotics followed by complete evaluation of CAKUT. EO in infants is almost always secondary to UTI. So, evaluate for UTI in all cases of acute EO and also for underlying CAKUT if EO is accompanied by complicated UTI. Testicular torsion is an organ-threatening condition. So, torsion should always be ruled out in an infant/child presenting with acute scrotum.
